# Extracts giving of purple eggplant (Solanum melongena L.) orally can lower blood serum levels of malondialdehide of white rat (Rattus novergicus) wistar diabetes mellitus induced by aloxan

**DOI:** 10.1186/1687-9856-2013-S1-O48

**Published:** 2013-10-03

**Authors:** Ellisma Swandini Nugraheni, Harjoedi Adji Tjahjono

**Affiliations:** 1 Faculty of Medicine, Brawijaya University, Malang, Indonesia

## 

Purple eggplant (Solanum melongena L.) is known to contain anthocyanins, which is about 850 mg/kg of eggplant. Anthocyanins are water soluble pigments which the antioxidant ability arising from structural conjugated bonding system, so having a high reactivity as a primary antioxidant, oxygen scavengers, and chelator, that these substances are expected to reduce oxidative stress in diabetes melitus (DM). This study aims to determine that the eggplant extract can reduce level of malondialdehyde (MDA) in the Wistar rat strain-aloxan induced model of diabetes mellitus and effective dose. This study is an experimental research with the design of the Control Group Post Test Only Design. The sample consisted of 25 rats that were divided into 5 groups, namely a negative control group (PO), a positive control group (PA) and 3 treatment groups (P1, P2, P3). Positive control group and three treatment groups were injected aloxan intraperitoneally as much as 150 mg/kg. Over the next 14 days, the treatment groups are administered the extract of purple eggplant with a dose of P1 = 343 mg/kg/hr, P2 = 686 mg/kg/hr and P3 = 1372 mg/kg/hr. Levels of MDA was measured after the last day of the extract by using UV-Vis specthrophotometer. Data were analyzed by ANOVA test with p ≤ 0.05. The results showed that they were significant differences in serum MDA levels between PO with the PA, as well as between the PA with all three treatment groups. Dose P1 are not significant lower levels of serum MDA, whereas P2 and P3 lower serum MDA levels significantly. There is inversely proportional powerful correlation between the dose of purple eggplant extract with MDA level with r = 0.761 and p = 0.000.

